# Interleukin-7 Induces Osteoclast Formation *via* STAT5, Independent of Receptor Activator of NF-kappaB Ligand

**DOI:** 10.3389/fimmu.2017.01376

**Published:** 2017-10-20

**Authors:** Jin-Hee Kim, Ji Hyun Sim, Sunkyung Lee, Min A. Seol, Sang-Kyu Ye, Hyun Mu Shin, Eun Bong Lee, Yun Jong Lee, Yun Jung Choi, Wan-Hee Yoo, Jin Hyun Kim, Wan-Uk Kim, Dong-Sup Lee, Jin-Hong Kim, Insoo Kang, Seong Wook Kang, Hang-Rae Kim

**Affiliations:** ^1^Department of Anatomy and Cell Biology, Seoul National University College of Medicine, Seoul, South Korea; ^2^Department of Biomedical Laboratory Science, College of Health Science, Cheongju University, Cheongju, South Korea; ^3^Department of Biomedical Sciences, Seoul National University College of Medicine, Seoul, South Korea; ^4^BK21Plus Biomedical Science Project, Seoul National University College of Medicine, Seoul, South Korea; ^5^Department of Pharmacology, Seoul National University College of Medicine, Seoul, South Korea; ^6^Medical Research Institute, Seoul National University College of Medicine, Seoul, South Korea; ^7^Department of Internal Medicine, Seoul National University College of Medicine, Seoul, South Korea; ^8^Department of Internal Medicine, Chonbuk National University Medical School and Research Institute of Clinical Medicine of Chonbuk National University Hospital, Jeonju, South Korea; ^9^Department of Internal Medicine, Chungnam National University School of Medicine, Daejeon, South Korea; ^10^Department of Internal Medicine, The Catholic University of Korea, Seoul, South Korea; ^11^Department of Biological Sciences, College of Natural Sciences, Seoul National University, Seoul, South Korea; ^12^Department of Internal Medicine, Section of Rheumatology, Yale University School of Medicine, New Haven, CT, United States

**Keywords:** osteoclast, intereleukin-7, IL-7 receptor alpha, STAT5, RANKL, monocyte

## Abstract

Interleukin-7 (IL-7), which is required for the development and survival of T cells in the thymus and periphery, plays a role in joint destruction. However, it remains unclear how IL-7 affects osteoclast formation. Thus, we investigated the mechanism by which IL-7 induced osteoclast formation through IL-7 receptor α (IL-7Rα) in osteoclast precursors. We cultured peripheral blood mononuclear cells or synovial fluid mononuclear cells with IL-7 in the presence or absence of an appropriate inhibitor to analyze osteoclast formation. We also constructed IL-7Rα-expressing RAW264.7 cells to uncover the mechanism(s) by which IL-7 induced osteoclast formation differed from that of receptor activator of nuclear factor κB ligand (RANKL). We found that IL-7 induced osteoclast formation of human monocytes from peripheral blood or synovial fluid in a RANKL-independent and a signal transducer and activator of transcription 5 (STAT5)-dependent manner. IL-7-induced osteoclasts had unique characteristics, such as small, multinucleated tartrate-resistant acid phosphatase positive cells and no alterations even when RANKL was added after IL-7 pretreatment. RAW264.7 cells, if overexpressing IL-7Rα, also were able to differentiate into osteoclasts by IL-7 through a STAT5 signaling pathway. Furthermore, IL-7-induced osteoclast formation was repressed by inhibitors of the IL-7R signaling molecules Janus kinase and STAT5. Our findings demonstrate that IL-7 is a truly osteoclastogenic factor, which may induce osteoclast formation *via* activation of STAT5, independent of RANKL. We also suggest the possibility that an IL-7R pathway blocker could alleviate joint damage by inhibiting osteoclast formation, especially in inflammatory conditions.

## Introduction

Bone is a dynamic tissue that changes its overall shape in response to physiological influences and mechanical forces, in a process called bone remodeling. Some pathological conditions lead to abnormal bone remodeling and an imbalance between bone resorption and formation that can develop into bone disorders [*reviewed in* Ref. (1)]. In particular, osteoclasts, in both normal and pathological conditions, originate from the hematopoietic (monocyte/macrophage) lineage, which then fuse to form active resorbing cells [*reviewed in* Ref. (2)]. In various inflammatory conditions, receptor activator of nuclear factor κB ligand (RANKL) binds to its receptor (RANK) on osteoclast precursors and serves as an essential factor in osteoclast formation, ultimately participating in the regulation of bone remodeling; this ligand is counterbalanced by osteoprotegerin (OPG) (3). RANKL is expressed by stromal cells, bone-lining cells, osteoblasts, and activated T cells [*reviewed in* Ref. (4)]. Interestingly, interleukin-1β (IL-1β) and tumor necrosis factor-α (TNF-α), which increase under pathological conditions, such as rheumatoid arthritis (RA) and osteoporosis, induce the expression of RANKL in osteoblasts and stromal cells, eventually enhancing osteoclast formation (5, 6).

Interleukin-7 (IL-7) is known to be a major player in the generation and maintenance of memory CD8^+^ T cells because it promotes cell survival even in the absence of antigen (7, 8). IL-7 is largely produced by stromal cells in the lymphoid tissue, intestinal epithelial cells, endothelial cells, fibroblasts, and following stimulation with IL-1β and TNF-α, by stromal cells [*reviewed in* Ref. (8, 9)]. IL-7 binds to its receptor, which consists of two components: a high-affinity IL-7Rα and a common gamma (γc) chain (10), and activates two pathways: the Janus kinase (JAK)/signal transducers and activator of transcription (STAT) and phosphoinositide-3 kinase (PI3K)/Akt, which lead to the development and survival of T cells (11).

However, IL-7 also induces bone loss *in vivo* (12, 13), stimulating osteoclast formation by enhancing the production of TNF-α and RANKL by T cells (14–16). In addition, levels of IL-7 correlate with disease severity and are increased in several arthritic conditions, such as RA (17–19). Although IL-7Rα is expressed mainly by lymphocytes and innate lymphoid cells, such as NK cells (20, 21), expression of IL-7Rα is elevated in the synovial tissues and macrophages from RA synovial fluid compared with macrophages from healthy controls, undifferentiated arthritis patients, and osteoarthritis patients (22, 23).

Thus, we hypothesized that IL-7 could directly induce osteoclast formation through its receptor IL-7Rα and its own signaling mechanism in precursor cells without RANKL. We cultured peripheral blood mononuclear cells (PBMCs) or synovial fluid mononuclear cells (SFMCs) with IL-7 in the presence or absence of an appropriate inhibitor to analyze osteoclast formation. We also constructed IL-7Rα-expressing RAW264.7 cells to uncover the mechanism by which IL-7 induces osteoclast formation that differs from that by RANKL.

## Materials and Methods

### Human Subjects

This protocol was approved by the Institutional Review Board of Seoul National University Hospital (#1406-043-584). Human peripheral blood and synovial fluid were drawn from healthy volunteers (24) and patients with RA after obtaining written informed consent in accordance with the Declaration of Helsinki.

### Cell Culture

Peripheral blood mononuclear cells in heparinized peripheral blood and SFMCs in heparinized joint fluid were purified using a Ficoll-Histopaque gradient (1.077 g/mL; GE Healthcare Bio-Sciences, Piscataway, NJ, USA). CD14^+^ monocytes were enriched from SFMCs, which was possible due to the monocytes’ ability to stick to the surface of tissue culture dishes. Briefly, SFMCs were plated on culture dishes for 1 h, then detached using 0.02% EDTA in cold phosphate-buffered saline. The purification of CD14^+^ monocytes was >95%, as confirmed by flow cytometry. PBMCs, SFMCs, and purified CD14^+^ monocytes were grown in α-minimum essential media (MEM) containing 10% fetal bovine serum (FBS) and 1% penicillin/streptomycin (i.e., α-MEM complete medium, all from Life Technologies, Carlsbad, CA, USA).

RAW264.7 cells were obtained from the American Type Culture Collection (ATCC, Manassas, VA, USA) and grown in Dulbecco’s modified Eagle’s medium (DMEM, Life Technologies) containing 10% FBS and 1% penicillin/streptomycin (i.e., DMEM complete medium). For osteoclast differentiation, RAW264.7 cells were cultured in α-MEM complete medium.

### Flow Cytometry

The following antibodies (Abs) were used for flow cytometry staining: phycoerythrin–anti-CD14 (BD Biosciences, San Jose, CA, USA), allophycocyanin-anti-IL-7Rα (eBioscience, San Diego, CA, USA), Alexa Fluor 700–anti-CD3 (eBioscience), and fluorescein isothiocyanate–anti-phospho-STAT5 (p-STAT5) (BD Biosciences). To analyze IL-7Rα expression, cells were stained with Abs to CD3, CD14, IL-7Rα or isotype control. To measure intracellular p-STAT5, cells were first stained with Abs to surface antigens and fixable viability dye eFluor 506 (eBioscience) to irreversibly label dead cells, then stimulated for 30 min with recombinant human IL-7 (10 ng/mL, PeproTech, Rocky Hill, NJ, USA) or PBS. The cells were then fixed with 2% paraformaldehyde for 10 min and permeabilized with 90% methanol for 30 min. Subsequently, the cells were stained with an Ab to p-STAT5. The stained cells were analyzed on a BD™ LSRII (BD Biosciences) with the FACSDiva™ software. After gating on CD3^+^ or CD14^+^ cells from live cells, expression levels of IL-7Rα and p-STAT5 were analyzed in T cells and monocytes.

### Plasmid Construction and Lentiviral Transduction

Interleukin-7Rα-overexpressing RAW264.7 cells (i.e., RAW-IL7ROE cells) were established using lentiviral vectors (Clontech Laboratories; Mountain View, CA, USA). cDNA for *IL7RA* was amplified from a cDNA clone using PCR (see Table S1 in Supplementary Material for primer sequences). The PCR product was cloned into the pLVX-IRES-ZsGreen1 vector, the destination lentiviral vector for the overexpression of IL-7Rα.

Lentiviruses were produced by co-transfection of 293FT cells (Life Technologies) with the cloned lentiviral vector, together with three packaging plasmids (pLP1, pLP2, pLP/VSVG) using PromoFectin (Promokine, Heidelberg, Germany). Then, the viruses were collected and concentrated using PEG-it (System Biosciences, Palo Alto, CA, USA). Cells were infected with the cloned lentivirus in the presence of 8 µg/mL hexadimethrine bromide (Life Technologies) by centrifugation (1,200 × *g*, 1 h, 32°C). To collect lentiviral-transduced cells, cells expressing a GFP reporter were sorted using a BD FACSAria system (BD Biosciences).

### Differentiation of Osteoclasts

Cells were cultured in 384-well culture plates in the presence of recombinant human macrophage colony-stimulating factor (M-CSF) (20 ng/mL), recombinant human RANKL (50 ng/mL), or IL-7 (2 ng/mL; all from PeproTech) for the indicated number of days by replacing the medium more than 90% of the original medium at 3-day intervals with fresh cytokines, thereby removing the effect of previously produced cytokines and unattached cells, including T and B cells to leave only adherent osteoclast precursor cells. Otherwise, cells were cultured with IL-7 (2 ng/mL) for 3 days, followed by treatment with M-CSF (20 ng/mL), RANKL (50 ng/mL), or IL-7 (2 ng/mL) by replacing the medium, as described above, to evaluate the effect of IL-7 pretreatment. OPG was purchased from PeproTech to inhibit RANKL/RANK signaling. The JAK inhibitor, tofacitinib, and a STAT5 inhibitor (CAS 285986-31-4; Sigma-Aldrich, St. Louis, MO, USA) were used to evaluate JAK/STAT signaling.

After 5–15 days of culture, cells were fixed and stained for tartrate-resistant acid phosphatase (TRAP) using an acid phosphatase, leukocyte kit (Sigma-Aldrich) according to the manufacturer’s protocol. Mature osteoclasts, which were defined as multinucleated (≥3 nuclei) TRAP^+^ cells, were counted manually with a light microscope (Olympus, Tokyo, Japan). Images were captured using the ProgRes Capture Pro software (Jenoptik, Jena, Germany).

Wild-type STAT5a (STAT5A-WT), dominant-negative STAT5a/b (STAT5A-DN, STAT5B-DN), and a constitutively active STAT5a (STAT5A-CA) (25) were transiently transfected into RAW274.7 or RAW-IL7ROE cells using electroporation (Bio-Rad, Hercules, CA, USA). Then, cells were cultured with recombinant mouse RANKL (50 ng/mL) in the presence or absence of recombinant mouse IL-7 (2 ng/mL) for 6 days by changing the medium at 3-day intervals with fresh cytokines. At day 6, cells were stained with TRAP.

### Pits Formation Assay

The functional resorption activity of the differentiated osteoclasts was evaluated by resorption pit formation, as described below. Cells were cultured for 30 days on top of dentine disks (Immunodiagnostic Systems, Boldon, UK) in 96-well culture plates in the presence of M-CSF (20 ng/mL), RANKL (50 ng/mL), or IL-7 (2 ng/mL) to differentiate osteoclasts. Then, the dentine slices were washed three times and immersed in 70% sodium hypochlorite to remove adherent cells. The resorption lacunae were counterstained with 1% (w/v) toluidine blue in 0.5% sodium borate for 60 s (Sigma-Aldrich). Photographs were taken through an LSM 5 PASCAL confocal microscope (Carl Zeiss, Jena, Germany) to analyze the surface topography and the area of the resorption pits was measured in four randomly selected areas for each dentine slice with the LSM 5 Image Browser (Carl Zeiss). Roughness parameters obtained were:*roughness average* (Ra), which is the main height calculated over the entire measured length or area, Rq, *statistical moments of peak distribution (symmetry)*, Rz, *mean roughness depth*, and Rv, *maximum profile valley depth*, which are the distances from the mean line/surface to the highest/lowest point in the evaluation length/area (26).

### Quantitative RT-PCR

For quantitative PCR (qPCR), total RNA was extracted from cells using TRIzol reagent (Life Technologies). Extracted RNA was used for cDNA synthesis using a Transcript First Strand cDNA synthesis Kit (Roche Applied Science, Basel, Switzerland). PCR was performed using qPCR PreMix (Bioneer, Daejeon, Republic of Korea) (see Table S1 in Supplementary Material for primer sequences). qPCR was performed with an Exicycler 96 Quantitative Real-Time PCR System (Bioneer). Differences in expression were normalized to expression of the control gene glyceraldehyde 3-phosphate dehydrogenase (*GAPDH*) or ribosomal protein S18 (*RPS18*).

### Immunoblotting Analysis

Protein lysate was resolved by 12% sodium dodecyl sulfate-polyacrylamide gel electrophoresis and transferred to a polyvinylidene difluoride membrane. The following Abs were used for immunoblotting: rabbit monoclonal Abs to phospho (p)-Akt (Ser473), Akt, extracellular signal-regulated kinases (Erk), p-Erk (Thr202/Tyr204) (all from Cell Signaling Technology, Danvers, MA, USA), p-STAT5 (EMD Millipore, Billerica, MA, USA), and STAT5 (BD Biosciences). The membranes were stained with appropriate Abs and visualized using SuperSignal West Femto Chemiluminescent Substrate (Thermo Fisher Scientific, Fremont, CA, USA).

### Statistical Analysis

All data are expressed as means ± SEM. Data were compared with the control group using a two-tailed Student’s *t*-test and two-way analysis of variance with Bonferroni *post hoc* test. *p* values <0.05 were considered to indicate statistical significance. All statistical analyses were performed using the GraphPad Prism software (ver. 6.01; GraphPad Software, La Jolla, CA, USA).

## Results

### IL-7 Induces Osteoclast Formation from PBMCs and SFMCs with Unique Characteristics

**Figure 1:**
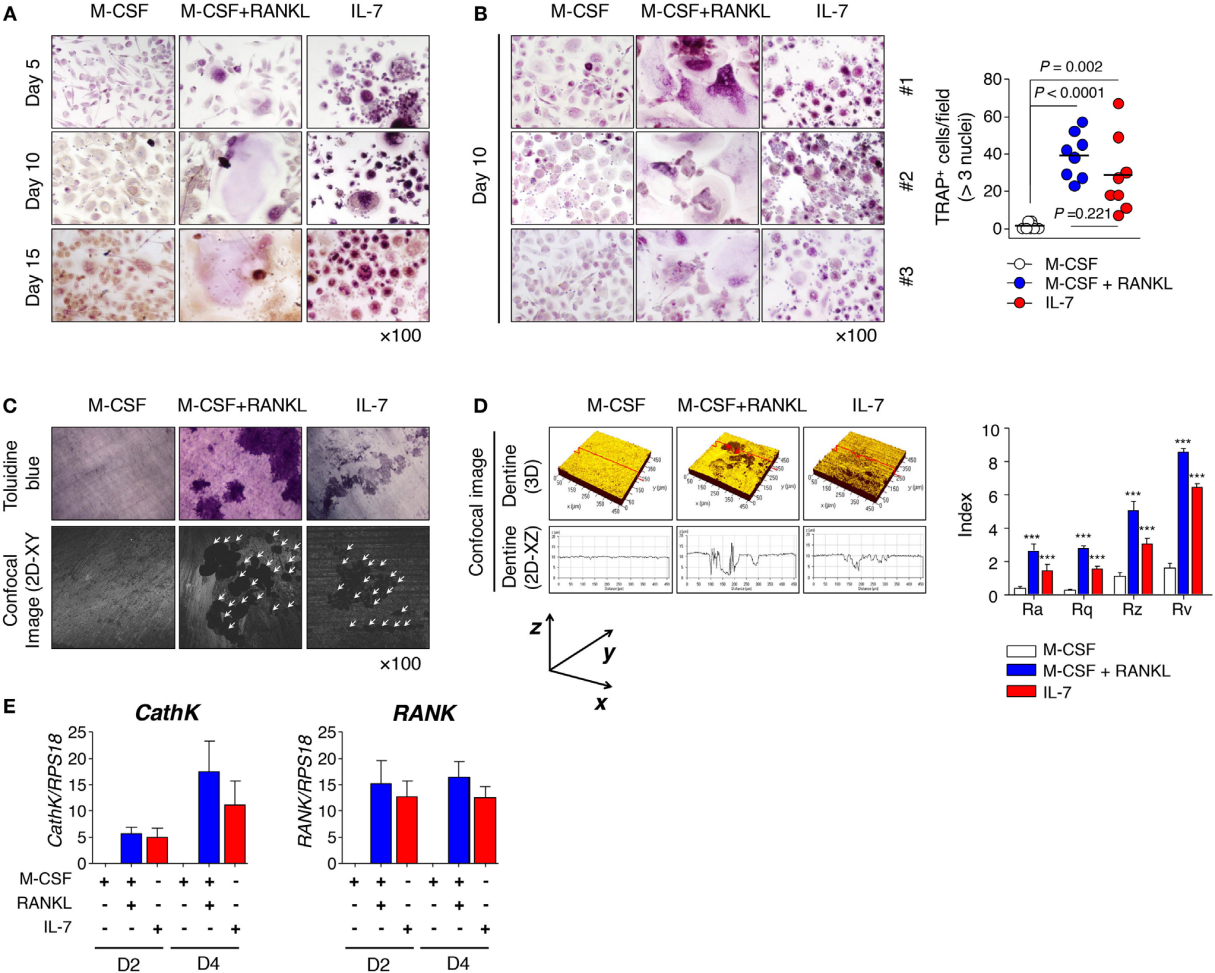
Interleukin (IL)-7 induced osteoclast formation in peripheral blood mononuclear cells (PBMCs) from healthy individuals. PBMCs from healthy individuals were treated with macrophage colony-stimulating factor (M-CSF; 20 ng/mL), receptor activator of nuclear factor κB ligand (RANKL; 50 ng/mL), or IL-7 (2 ng/mL) for the indicated periods by replacing the medium at 3-day intervals with fresh cytokines. Cells were fixed and stained for tartrate-resistant acid phosphatase (TRAP). **(A,B)** Mature osteoclasts, which were defined as multinucleated (≥3 nuclei) TRAP^+^ cells, were counted manually using a light microscope. Representative images **(A,B)** and quantification **(B)** showing TRAP^+^ cells are shown. Results are representative of five independent experiments with eight different donors. Bars represent the mean and *p* values were obtained using the unpaired two-tailed Student’s *t*-test. **(C)** PBMCs were cultured for 30 days on top of dentine disks in 96-well culture plates in the presence of M-CSF (20 ng/mL), RANKL (50 ng/mL), or IL-7 (2 ng/mL). After removing adherent cells, the resorption lacunae were counterstained with toluidine blue. Photographs were taken through a confocal microscope and the area of resorption pits was measured in four randomly selected areas for each dentine slice. Representative toluidine blue image (upper panel) and confocal image (lower panel) are shown. Data are representative of three independent experiments (*n* = 3). The arrowhead indicates a resorption pit. **(D)** Roughness parameters [roughness average (Ra) calculated over the entire measured length or area, statistical moments of peak distribution (symmetry, Rq), mean roughness depth (Rz), and maximum profile valley depth (Rv)] and the number of pits are indicated. The graph represents the mean ± SEM and *p*-values were obtained using two-way analysis of variance followed by Bonferroni *post hoc* test. ***p < 0.001, vs. the control group. **(E)** PBMCs were cultured for the indicated number of days with M-CSF (20 ng/mL), RANKL (50 ng/mL), or IL-7 (2 ng/mL). Then, quantitative RT-PCR of cathepsin K (*CathK*) and *RANK* was performed and the data was normalized to ribosomal protein S18 (*RPS18*). The graph represents the mean ± SEM (*n* = 9).

Although IL-7 is known to stimulate osteoclast formation by augmenting the production of TNF-α and RANKL by T cells (14–16, 27), there has been little research on the characteristics of osteoclasts induced by IL-7 or the characteristics of progenitor cells derived under inflammatory conditions. Thus, we explored how IL-7 affected osteoclast formation by analyzing the pattern of multinucleated TRAP^+^ cells (≥3 nuclei), and measuring roughness using various parameters for dentine pits. PBMCs from healthy individuals and SFMCs from patients with RA were cultured for 15 or 30 days with M-CSF, RANKL, and IL-7 to determine osteoclast differentiation. We found that osteoclasts from PBMCs whose differentiation was induced by M-CSF and RANKL were typically giant multinucleated TRAP^+^ cells by day 10 (Figures 1A,B). However, IL-7-mediated TRAP^+^ cells were small multinucleated cells that appeared even earlier, on day 5 (Figure 1A), a differentiation that was similarly observed in SFMCs (Figure S1A in Supplementary Material). On day 10, however, osteoclast differentiation from PBMCs by IL-7 was comparable to that from M-CSF and RANKL (Figure 1B), while osteoclast formation from SFMCs by IL-7, although meaningful compared to the M-CSF control, was significantly lower than that induced by M-CSF and RANKL (Figure S1B in Supplementary Material). Overall, osteoclast formation from SFMCs was superior to PBMCs in terms of the number of TRAP^+^ cells. Although the size of the multinucleated TRAP^+^ cells by IL-7 was small, we found that the roughness of dentine pit formation by IL-7-induced osteoclasts was comparable with the pit formation by M-CSF- and RANKL-induced osteoclasts, regardless of whether the osteoclasts were derived from PBMCs or SFMCs (Figures 1C,D; Figures S1C,D in Supplementary Material). That is, when considering roughness parameters—Ra, Rq, Rz, and Rv—the depth of the pits formed by the IL-7, M-CSF, and RANKL-induced osteoclasts were equivalent (Figure 1D; Figure S1D in Supplementary Material) although the area of the pits, as shown by toluidine blue staining, was larger for M-CSF and RANKL than for IL-7 (Figure 1C; Figure S1C in Supplementary Material). Next, we further analyzed the expression of osteoclast markers [cathepsin K (*CathK*) and *RANK*] induced by IL-7, M-CSF, and RANKL. IL-7 induced the expression of *CathK* and *RANK* comparable to M–CSF and RANKL (Figure 1E; Figure S1E in Supplementary Material), suggesting that IL-7 can induce osteoclast differentiation through a mechanism similar to RANKL.

We have demonstrated that IL-7-mediated osteoclasts are unique in TRAP^+^ cell size and functional resorption, such as dentine pit formation. These findings suggest that IL-7-induced osteoclast formation might occur by a mechanism different from the previously known mechanisms involving the induction of TNF-α and RANKL by T cells (14–16).

### IL-7 Determines the Fate of Osteoclast Differentiation Early on and Dominantly Preserves the Characteristics of Osteoclasts by IL-7

**Figure 2:**
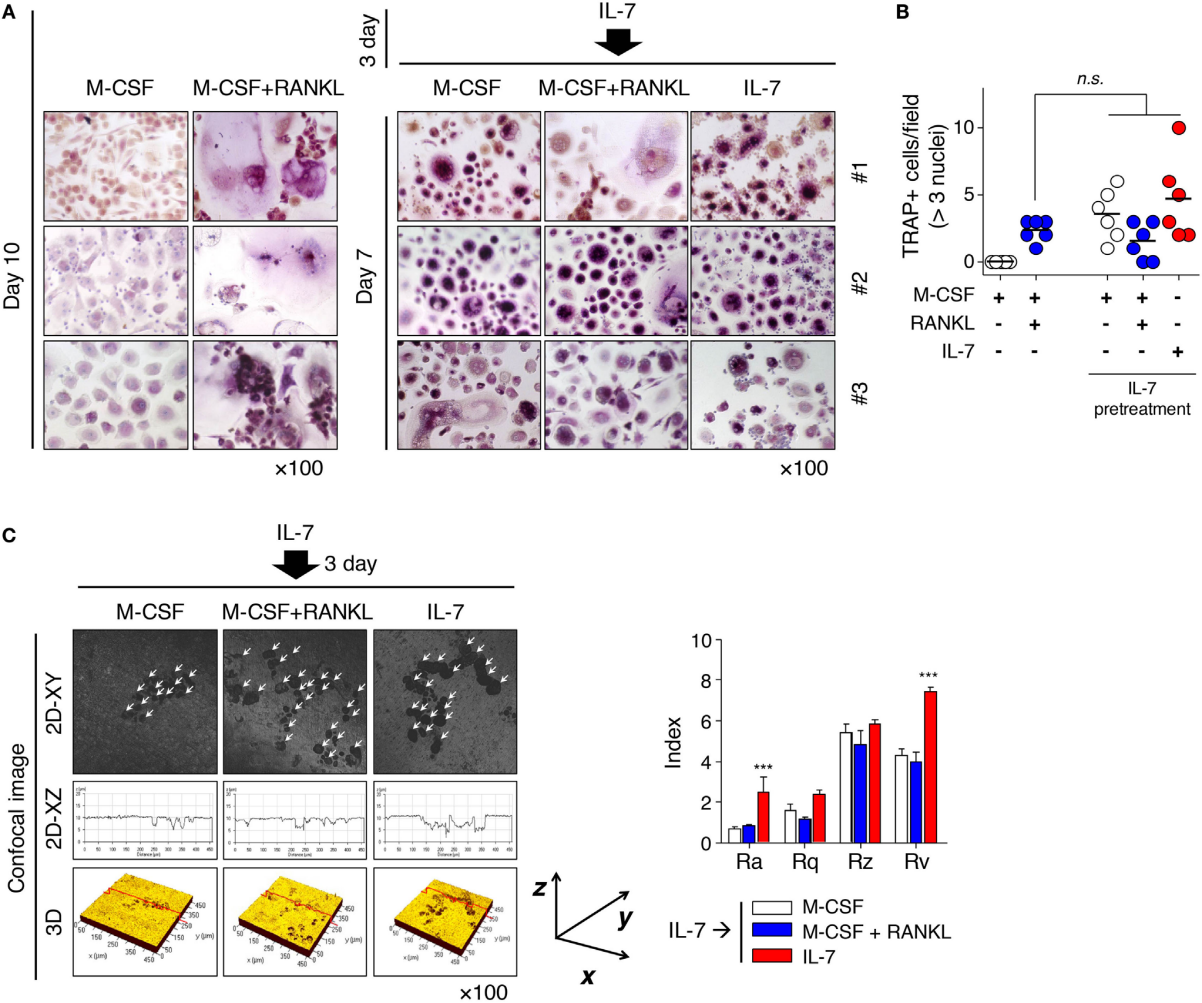
Interleukin (IL)-7 induced osteoclast formation in synovial fluid mononuclear cells (SFMCs) from joint fluid of rheumatoid arthritis (RA) patients. SFMCs were cultured with M-CSF (20 ng/mL), RANKL (50 ng/mL), or IL-7 (2 ng/mL) for 10 days by replacing the medium at 3-day intervals with fresh cytokines as described in Figure 1 (left panel). To determine the effect of pretreatment with IL-7, SFMCs were cultured with IL-7 (2 ng/mL) for 3 days, then treated with M-CSF (20 ng/mL), RANKL (50 ng/mL), or IL-7 (2 ng/mL) for 7 days, replacing the medium as described above (right panel). TRAP staining and enumeration were performed as described in Figure 1. Representative images **(A)** and quantification **(B)** of TRAP^+^ cells at days 10 and 15 are shown. Results are representative of five independent experiments with five different donors. Bars represent the mean and *p* values were obtained using the unpaired two-tailed Student’s *t*-test. **(C)** Peripheral blood mononuclear cells were cultured on top of dentine disks in 96-well culture plates in the above condition for 30 days. Then, surface roughness was analyzed as described in Figure 1. Results illustrate three independent experiments (*n* = 3). Roughness parameter and the number of pits were analyzed as described in Figure 1. The graph represents the mean ± SEM and *p* values were obtained using the unpaired two-tailed Student’s *t*-test.

These results raised a question about whether the fate of IL-7-induced osteoclasts could be altered by the osteoclastogenic factor, RANKL, because osteoclasts formed by IL-7 differed from those by RANKL in many ways (Figure 1; Figure S1 in Supplementary Material). To address this, we pretreated cells with IL-7, then treated them with M-CSF, RANKL, or IL-7, replacing more than 90% of the original medium at 3-day intervals to remove the effect of previously produced cytokines and unattached cells. Through preliminary experiments, we found that most T and B cells were removed by the third day of media replacement (*data not shown*), which suggested that in our experimental conditions, the removal would take 3 days even if IL-7 acts on T cells, as reported previously (14). Surprisingly, the small, multinucleated TRAP^+^ cells of IL-7-induced osteoclasts (Figure 1; Figure S1 in Supplementary Material) were not altered when RANKL was added after IL-7 pretreatment (Figure 2A). However, IL-7 pretreatment induced osteoclast formation to a level similar to that of RANKL treatment (Figure 2B), and the fully differentiated osteoclasts formed dentine pits (Figure 2C). Interestingly, after IL-7 pretreatment, M-CSF alone resulted in the formation of TRAP^+^ cells, which were capable of forming dentine pits (Figures 2A,C). However, continuous treatment with IL-7 led to the formation of dentine pits deeper than those formed by cells treated with RANKL following IL-7 pretreatment (Figure 2C). This raised the possibility that IL-7 may act directly and uniquely on osteoclasts precursors, a notion that would be supported if IL-7Rα was expressed on osteoclast precursors.

### IL-7 Directly Induced Osteoclast Formation from IL-7Rα-Expressing CD14^+^ Monocytes, Enriched in Synovial Fluid

**Figure 3:**
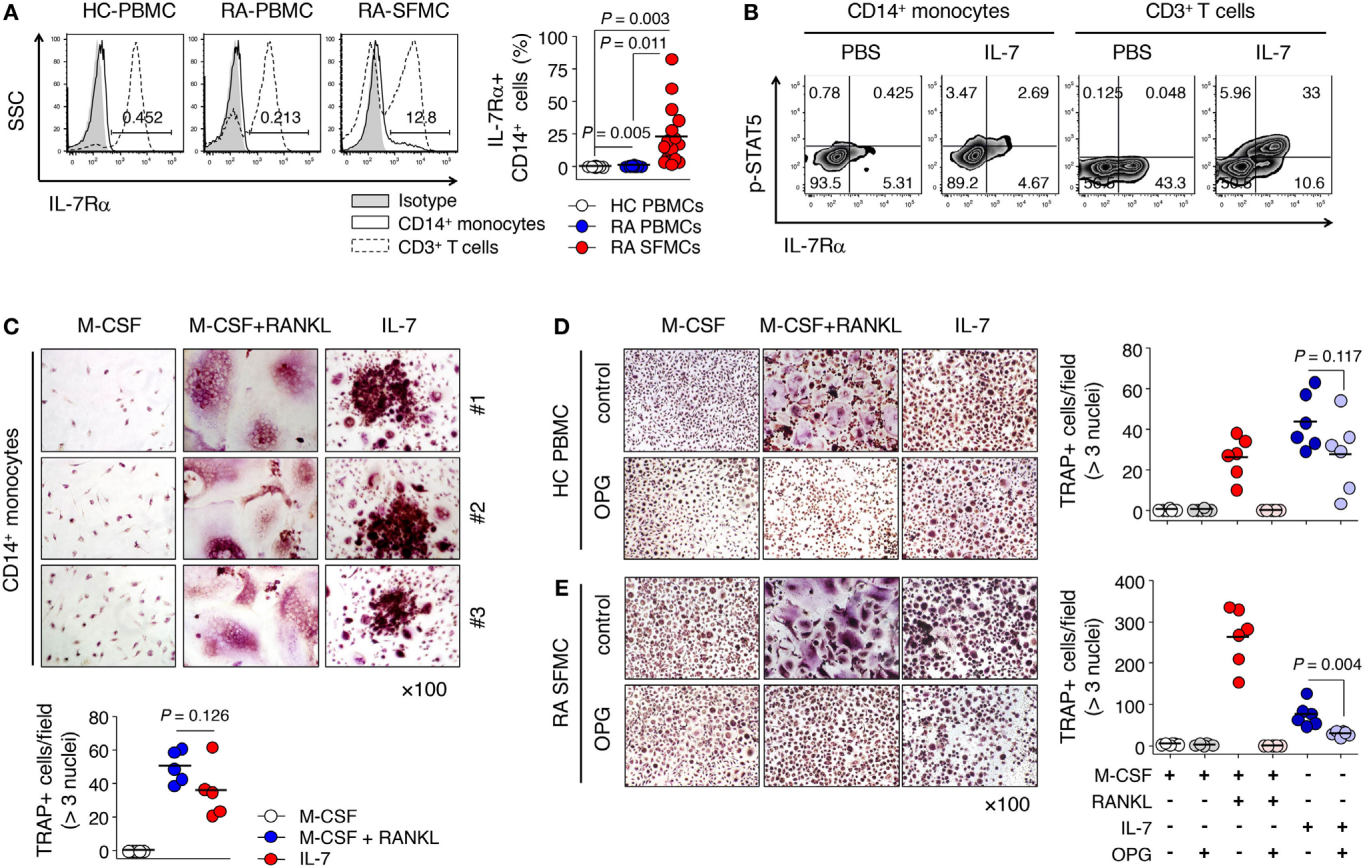
Interleukin (IL)-7 induced osteoclast formation from IL-7 receptor α (IL-7Rα)^+^ monocytes. **(A)** Cells were stained with antibodies (Abs) for CD3, CD14, and IL-7Rα expression was measured by flow cytometry. Representative histograms of IL-7Rα expression in CD14^+^ monocytes and CD3^+^ T cells (left panel) and the graph showing the frequencies of IL-7Rα expressing CD14^+^ monocytes [right panel, healthy (HC) peripheral blood mononuclear cells (PBMCs), *n* = 11, rheumatoid arthritis (RA) PBMCs, *n* = 8, RA synovial fluid mononuclear cells, *n* = 17]. Results are representative of six independent experiments with 8–17 different donors. Bars represent the mean and *p*-values were obtained using the unpaired two-tailed Student’s *t*-test. **(B)** Cells were stained with Abs for CD3, CD14, IL-7Rα, and fixable viability dye, then stained with Abs for p-signal transducer and activator of transcription 5 (p-STAT5) after incubation with IL-7 (10 ng/mL) or PBS for 30 min. Representative histogram of p-STAT5 and IL-7Rα. **(C)** CD14^+^ monocytes from patients with RA were cultured with M-CSF (20 ng/mL), RANKL (50 ng/mL), or IL-7 (2 ng/mL) for 10 days, replacing the medium at 3-day intervals with fresh cytokines. Tartrate-resistant acid phosphatase (TRAP) staining and enumeration were performed as described in Figure 1. Representative images and quantification of TRAP^+^ cells are shown. Results are representative of six independent experiments with six different donors. Bars represent the mean and *p* values were obtained using the unpaired two-tailed Student’s *t*-test. **(D,E)** PBMCs from healthy individuals **(D)** and SFMCs from RA patients **(E)** were cultured with M-CSF (20 ng/mL), RANKL (50 ng/mL), or IL-7 (2 ng/mL) in the presence or absence of osteoprotegerin (OPG; 100 ng/mL) for 10 days, replacing the medium at 3-day intervals with fresh cytokines and osteoprotegerin (OPG). TRAP staining and enumeration were performed as described in Figure 1. Representative images and quantification of TRAP^+^ cells are shown. Results are representative of six independent experiments with six different donors. Bars represent the mean and *p* values were obtained using the unpaired two-tailed Student’s *t*-test.

Based on our observations and previous reports (22, 23), we proposed the idea that IL-7Rα could be induced by inflammatory factors produced by T or B cells even if IL-7Rα is not expressed in progenitor cells. As expected, we found that the frequency of IL-7Rα^+^ CD14^+^ monocytes was increased significantly in the synovial fluid of RA patients compared with peripheral blood from both RA patients and healthy individuals, where IL-7Rα was rarely expressed (Figure 3A). Of particular importance, this increased IL-7Rα on monocytes reacted with IL-7 to phosphorylate STAT5 (Figure 3B), a finding that raised the possibility of direct osteoclast differentiation by IL-7 for precursor cells that express IL-7Rα. In particular, stimulation of PBMCs from healthy individuals with IL-7 resulted in increased expression of IL-7Rα, as well as STAT5 activation in monocytes (Figure S2 in Supplementary Material). This suggests that IL-7 acts on IL-7Rα-expressing cells first, thereby inducing functional IL-7Rα expression in CD14^+^ cells and ultimately stimulating osteoclast formation.

Next, to confirm whether IL-7 directly induced TRAP^+^ cells of IL-7Rα^+^ monocytes, we isolated CD14^+^ monocytes expressing IL-7Rα from SFMCs of RA patients and cultured them with IL-7. Remarkably, these CD14^+^ monocytes differentiated into TRAP^+^ cells with IL-7 (Figure 3C); the degree of osteoclast differentiation was similar to that with M-CSF and RANKL (Figure 3C). This finding demonstrates for the first time that IL-7 alone can induce sufficient osteoclast differentiation from human monocytes that express IL-7Rα.

Interleukin (IL)-7 has been shown to upregulate RANKL secretion in T cells (14), which led us to ask whether IL-7-induced osteoclast formation was due to the presence of secreted RANKL. To exclude this possibility, we added the RANKL inhibitor OPG, a naturally occurring secreted protein (28), into cultures of PBMCs and SFMCs treated with IL-7. We found that OPG completely inhibited RANKL-induced osteoclast formation (Figures 3D,E), but hardly blocked osteoclast formation from PBMCs by IL-7 (Figure 3D). However, OPG repressed IL-7-induced osteoclast formation from SFMCs by approximately 60% (Figure 3E; mean ± SEM = 75.2 ± 28.8 vs. 29.8 ± 6.2). Thus, OPG did not inhibit IL-7-induced osteoclast formation, although it partially blocked osteoclast differentiation from SFMCs, suggesting that IL-7 can directly induce osteoclast formation in a RANKL-independent manner.

### Osteoclast Differentiation by IL-7 Depends on the Activation of STAT5

**Figure 4:**
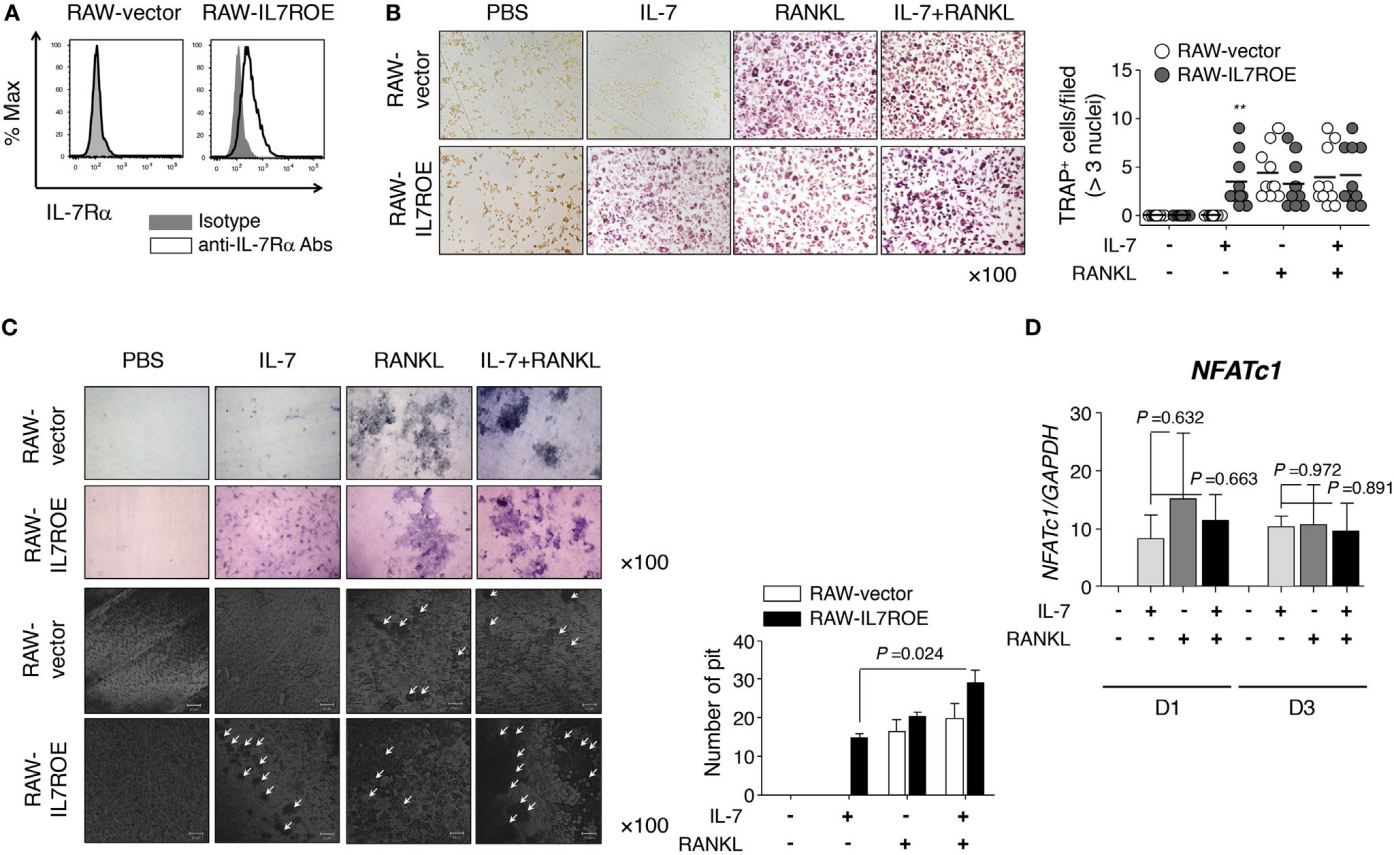
Interleukin (IL)-7 induced osteoclast formation in RAW264.7 cells expressing IL-7Rα. **(A)** RAW264.7 expressing IL-7Rα (RAW-IL7ROE) were established by lentiviral transduction and sorted using a BD FACSAria system. IL-7Rα expression on the surface of RAW-IL7ROE cells was measured by flow cytometry (IL-7Rα; open, isotype control; shaded). **(B)** Cells were cultured with RANKL (50 ng/mL) and/or IL-7 (2 ng/mL) for 6 days, replacing the medium at 3-day intervals with fresh cytokines. Tartrate-resistant acid phosphatase (TRAP) staining and enumeration were performed as described in Figure 1. Representative images and quantification of TRAP^+^ cells are shown. Results are representative of three independent experiments (*n* = 10). Bars represent the mean and *p* values were obtained using two-way ANOVA followed by Bonferroni *post hoc* test. **p < 0.01, vs. the control group (RAW-vector). **(C)** Cells were cultured on top of dentine disks in 96-well culture plates in the conditions above for 14 days. Then, the number of pits was analyzed as described in Figure 1. The arrowhead indicates the resorption pit. Results are representative of three independent experiments (*n* = 3). Graph represents the mean ± SEM and *p* values were obtained using the unpaired two-tailed Student’s *t*-test, for comparing IL-7 and IL-7 + RANKL-treated group in RAW-IL7ROE. **(D)** RAW-IL7ROE cells were cultured for the indicated number of days with RANKL (50 ng/mL) and/or IL-7 (2 ng/mL), replacing the medium at 3-day intervals with fresh cytokines. Then, quantitative RT-PCR of nuclear factor of activated T-cells 1 (*NFATc1*) was performed and the data was normalized with glyceraldehyde 3-phosphate dehydrogenase. Results are representative of two independent experiments (*n* = 2). Graph represents the mean ± SEM and *p*-values were obtained using the unpaired two-tailed Student’s *t*-test.

Based on our observations, we investigated the mechanism by which IL-7 induced osteoclast differentiation *via* IL-7Rα using RAW264.7 cells, a mouse leukemic monocyte-macrophage cell line. We constructed IL-7Rα-overexpressing RAW264.7 cells (RAW-IL7ROE) using lentiviral transduction (Figure 4A) and determined whether they differentiated into osteoclasts in response to IL-7. We found that IL-7 induced osteoclast differentiation of RAW-IL7ROE cells, but not control (RAW-vector) cells (Figure 4B). However, IL-7, in combination with RANKL, induced multinucleated TRAP^+^ cells in both RAW-IL7ROE and RAW-vector cells in comparable numbers (Figure 4B). IL-7 in combination with RANKL significantly increased the number of dentine pits formed by osteoclasts differentiated from RAW-IL7ROE cells compared with IL-7 alone (Figure 4C), suggesting that IL-7 could induce osteoclast formation by a different mechanism than RANKL. We also found that OPG completely inhibited RANK-induced TRAP^+^ cells, but did not affect the formation of TRAP^+^ cells by IL-7 or IL-7 in combination with RANKL (Figure S4 in Supplementary Material). Thus, these findings confirmed that IL-7 alone can cause differentiation into fully functioning osteoclasts.

To address the underlying mechanisms, we analyzed the expression of osteoclast markers [*TRAP, CathK*, tyrosine-protein kinase SRC-1 (*c-src*), and calcitonin receptor (*CalcR*)] induced by IL-7 and RANKL. Overall, IL-7 induced the expression of osteoclast marker genes at similar levels to RANKL (Figures S3A,B in Supplementary Material). Furthermore, we explored whether the expression of nuclear factor of activated T-cells 1 (*NFATc1*), a master regulator gene that is upregulated by RANKL, was affected by IL-7 during osteoclastogenesis because RANKL upregulates *NFATc1 via* the expression of the early osteoclastic gene, *c-Fos*, which, in turn, induces osteoclast marker genes (29). We found that IL-7 induced expression of *NFATc1* mRNA at a level comparable to that of RANKL, but there was no synergistic effect with RANKL (Figure 4D). Together, this suggests that IL-7 can induce the differentiation of functional osteoclasts similar to that of the osteoclastogenic factor RANKL.

**Figure 5:**
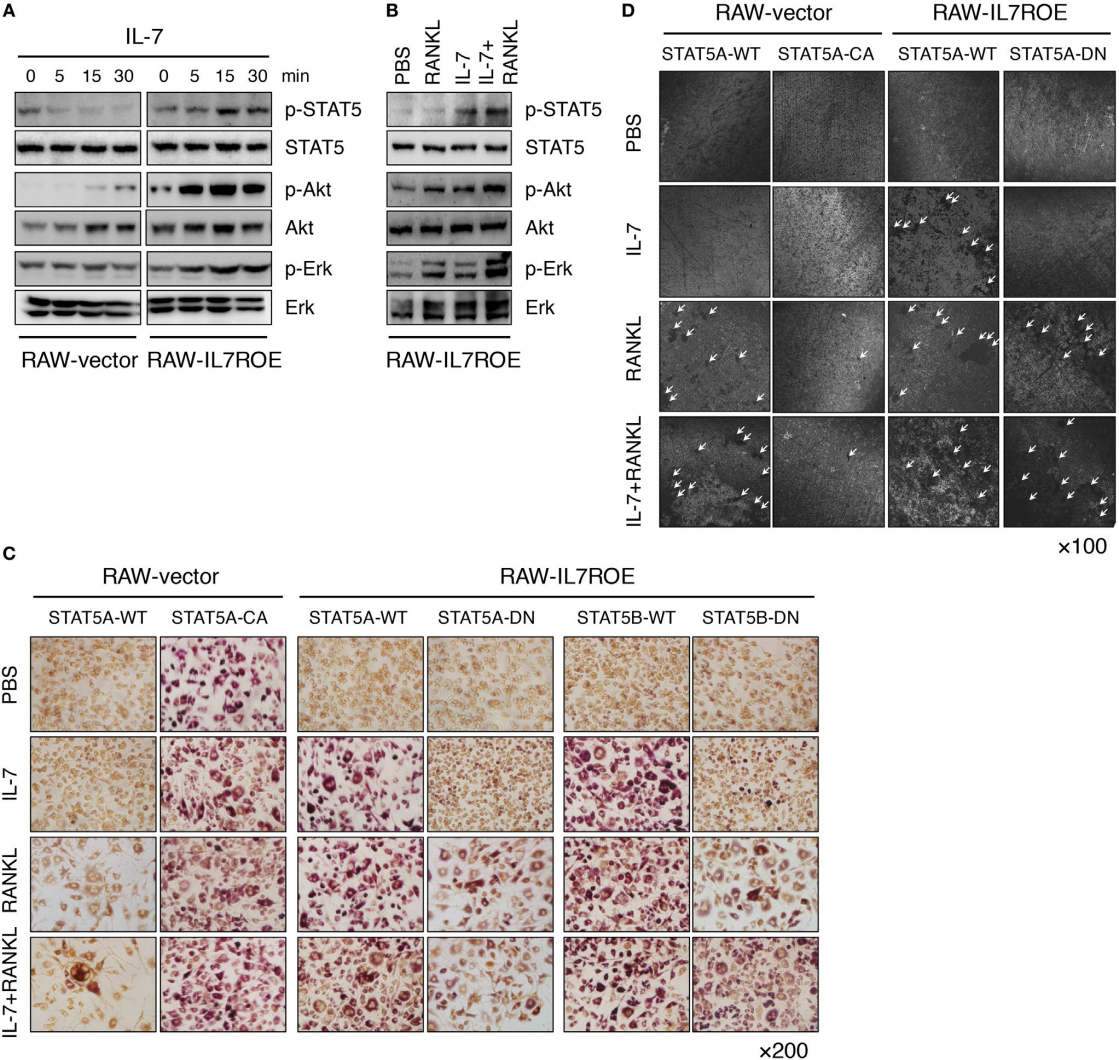
Interleukin (IL)-7 induced osteoclast formation through a signal transducer and activator of transcription 5 (STAT5) signaling pathway. **(A)** Control (RAW-vector) and RAW-IL7ROE cells were stimulated for the indicated time intervals with IL-7 (10 ng/mL). Then, immunoblot analysis was performed. Results are representative of three independent experiments. **(B)** RAW-IL7ROE cells were stimulated for 30 min with RANKL (50 ng/mL) and/or IL-7 (10 ng/mL). Then, immunoblot analysis was performed. Results are representative of two independent experiments. **(C)** RAW-vector or RAW-IL7ROE cells were transfected with wild-type STAT5a (STAT5A-WT), constitutively active STAT5a (STAT5A-CA), and dominant-negative STAT5a/b (STAT5A-DN, STAT5B-DN), then cultured for 6 days with RANKL (50 ng/mL) and/or IL-7 (2 ng/mL), replacing the medium at 3-day intervals with fresh cytokines. TRAP staining was performed as described in Figure 1. Representative images showing TRAP^+^ cells on day 6 are shown. Results are representative of two independent experiments. **(D)** Cells were cultured on top of dentine disks in 96-well culture plates in the above conditions for 14 days. Then, surface roughness was analyzed as described in Figure 1. Representative confocal images are shown. The arrowhead indicates the resorption pit. Results are representative of two independent experiments.

Finally, to explore how IL-7 induces osteoclast differentiation through IL-7Rα in progenitors in more detail, we analyzed components of early IL-7R signaling, including p-STAT5, p-Akt, and p-Erk, to determine which signaling molecules were uniquely activated by IL-7, and not by RANKL. We discovered that activation of Akt and Erk was common to RANKL and IL-7, whereas activation of STAT5 was unique to IL-7 (Figures 5A,B), suggesting that STAT5 could be a key molecule in IL-7-induced osteoclast formation.

Based on these observations, we transfected constitutively active STAT5a (STAT5A-CA) or dominant-negative STAT5a/b (STAT5A-DN or STAT5B-DN) plasmids into RAW-vector and RAW-IL7ROE cells to further analyze the role of STAT5 in IL-7-induced osteoclast formation. When RAW-IL7ROE cells were transfected with STAT5A-DN or STAT5B-DN, RANKL, or IL-7 in combination with RANKL-induced osteoclast formation, but IL-7 did not (Figures 5C,D). These findings indicate that STAT5 is a key molecule in IL-7-mediated osteoclast formation. Interestingly, while STAT5A-CA transfection alone was able to induce differentiation of multinucleated TRAP^+^ cells in the absence of stimulation (Figure 5C), it failed to induce dentine pit formation and even repressed dentine pit formation by RANKL (Figure 5D). These data suggest that despite the fact that the activation of STAT5 by IL-7 can produce functional osteoclasts that form dentine pits, inappropriate or excessive STAT5 activity in the absence of IL-7 may inhibit RANKL-induced osteoclast formation.

**Figure 6:**
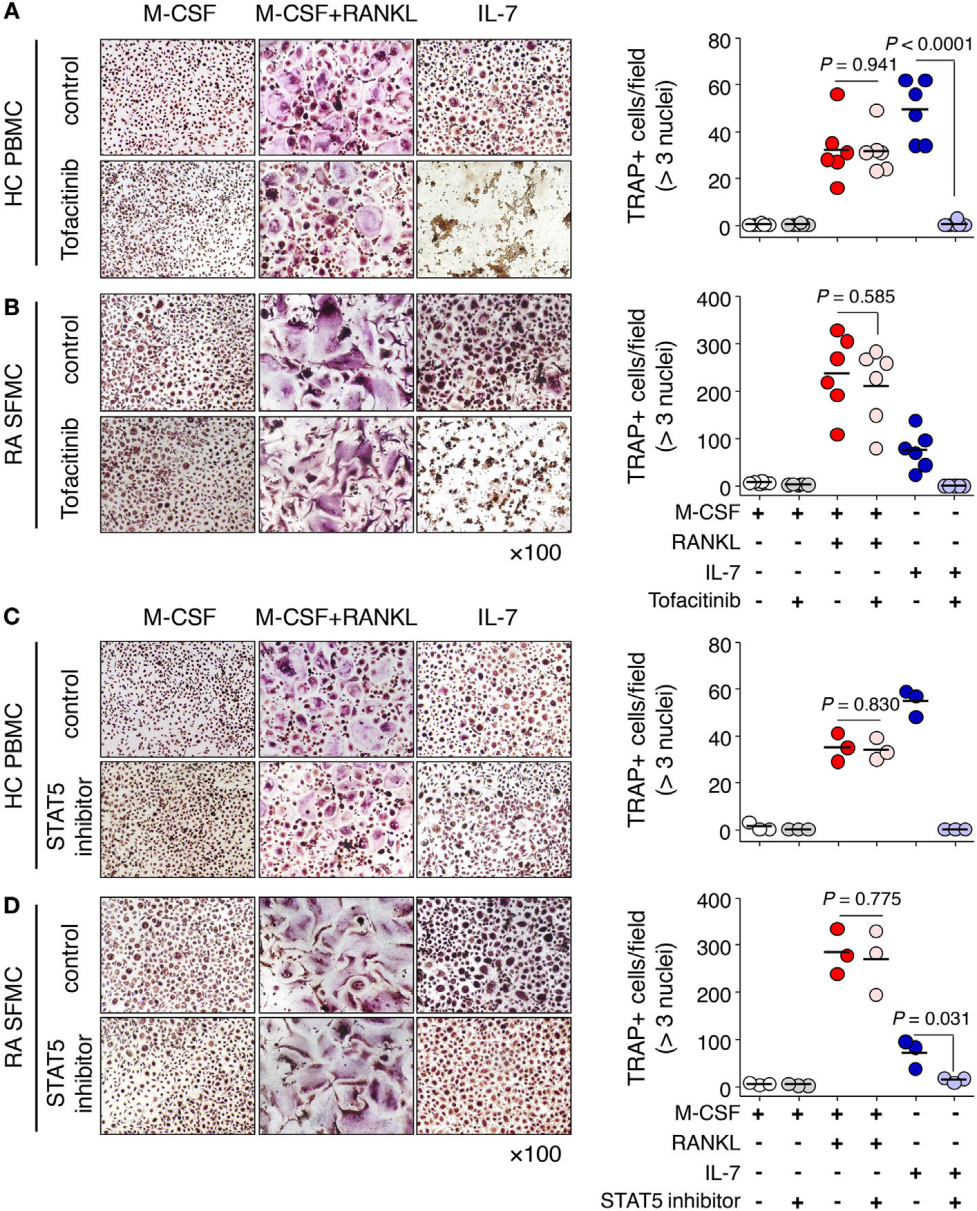
Janus kinase (JAK)/STAT inhibitors blocked Interleukin (IL)-7-mediated osteoclast formation from peripheral blood mononuclear cells (PBMCs) and synovial fluid mononuclear cells (SFMCs). PBMCs from healthy individuals **(A,C)** and SFMCs from rheumatoid arthritis patients **(B,D)** were cultured with M-CSF (20 ng/mL), RANKL (50 ng/mL), or IL-7 (2 ng/mL) in the presence or absence of tofacitinib [5 µM, **(A,B)**] or signal transducer and activator of transcription 5 (STAT5) inhibitor [CAS 285986-31-4, 50 µM, **(C,D)**] for 10 days, replacing the medium at 3-day intervals with fresh cytokines and osteoprotegerin. Tartrate-resistant acid phosphatase (TRAP) staining and enumeration were performed as described in Figure 1. Representative images and quantification of TRAP^+^ cells are shown. Results are representative of three or six independent experiments. Bars represent the mean and *p* values were obtained using the unpaired two-tailed Student’s *t*-test.

Finally, we explored the clinical utility of IL-7R signaling in IL-7-mediated osteoclast formation. To address this, we used the JAK inhibitor tofacitinib, which was the first drug found to interfere with the JAK-STAT signaling pathway (30), and a STAT5 inhibitor, which interferes with DNA binding of STAT5 (31). We found that tofacitinib markedly inhibited IL-7-induced osteoclast formation from PBMCs as well as SFMCs, but not by RANKL (Figures 6A,B). The STAT5 inhibitor also considerably suppressed osteoclast formation in response to IL-7 in both PBMCs and SFMCs (Figures 6C,D). Furthermore, IL-7-induced osteoclast formation through the JAK/STAT signaling pathway was again confirmed by inhibitor treatment in RAW-IL7ROE cells (Figure S4 in Supplementary Material). Together, these data suggest that the IL-7R signaling pathway may serve as a target for the treatment of bone erosion by IL-7.

## Discussion

Our findings provide the first successful direct identification of osteoclast differentiation and its mechanism by IL-7 through its receptor on osteoclast precursors. In particular, IL-7-induced osteoclasts were uniquely characterized by the formation of small, multinucleated TRAP^+^ cells, which could not be reversed by RANKL. We used IL-7Rα-overexpressing cells and discovered a direct osteoclastogenesis mechanism induced by IL-7, which occurred *via* STAT5 activation. Based on these findings, we propose a model for osteoclast differentiation by IL-7 that acts in a RANKL-independent and STAT5-dependent manner. JAK/STAT5 inhibitors significantly blocked IL-7-induced osteoclast formation. These results, along with the fact that the JAK inhibitor tofacitinib significantly reduced signs and symptoms of RA and inhibited the progression of structural joint damage, as compared with methotrexate (30), suggest the possibility of IL-7 as a therapeutic target, inhibiting IL-7-induced bone loss.

It is known that inflammatory cytokines, such as IL-1, IL-6, and TNF-α, act on joint inflammation and damage by inducing RANKL in synovial fibroblasts and eventually forming osteoclasts (6, 32, 33). In particular, the combination of TNF-α and stromal cell-derived factor-1α (34–37) or the combination of TNF-α and IL-6 (38) promote osteoclast differentiation, independent of RANKL. While denosumab, a RANKL-specific blocking Ab, successfully treats bone damage in patients with RA (39, 40), denosumab was not used in monotherapy but combined with various inflammatory medications (39) because neither denosumab nor bisphosphonate, despite its ability to significantly suppress bone erosion, is able to suppress inflammation or RA disease activity. In addition, it has been reported that the addition of OPG, a RANKL antagonist, could not completely abolish dentine resorption (41). It is also known that RANKL and OPG are highly expressed by proinflammatory cytokine-stimulated fibroblasts at the same time. This is interesting because of the fact that strong osteoclastogenic effects *via* increased RANKL expression are counteracted by elevated expression of OPG due to a complex with soluble RANKL (42). Together, these findings suggest that the best RA treatment would be the use of drugs that can prevent both joint erosion and inflammation.

In this regard, IL-7, particularly in an inflammatory milieu, appears to be an attractive target molecule. Elevated levels of IL-7 have been reported in several arthritic conditions, including RA (43). Furthermore, there is a close correlation between serum or synovial fluid IL-7 levels in RA patients and severity markers of the disease (19). In arthritic conditions, IL-7 promotes expansion of lymphocytes associated with Th1 and Th17 activity, augmenting the severity of the disease, including synovitis, pannus, and bone and cartilage erosion (13). It is also known that IL-7 promotes osteoclast formation by activating T cells *via* both RANKL-dependent and -independent mechanisms (14). Unlike denosumab, blocking IL-7R inhibits collagen-induced arthritis, reduces T cell activity, and is associated with proinflammatory mediators (44). Here, we demonstrated that IL-7 directly induced osteoclastogenesis through IL-7Rα on osteoclast precursors in a RANKL-independent manner, even though OPG partially repressed IL-7-induced osteoclast formation from SFMCs. Thus, we believe that IL-7, such as TNF-α, IL-6, and IL-1, is a potent factor that can itself induce osteoclasts, suggesting the potential of IL-7 as the “best” therapeutic target for alleviating arthritis by suppressing both inflammation and bone erosion.

Furthermore, we found that RAW-vector cells, lacking IL-7Rα expression and transfected with STAT5-CA, differentiated into multinucleated TRAP^+^ cells but did not markedly form dentine pits regardless of any stimulus (Figures 5C,D). This result was confusing because it seemed to be contrary to the result that IL-7 induced osteoclast formation in a STAT5-dependent manner, which was blocked by a STAT5 inhibitor (Figures 6C,D; Figure S4 in Supplementary Material). However, recently Lee et al. (45) and Hirose et al. (46) reported that IL-3-induced activation of STAT5A or STAT5B can sufficiently inhibit bone resorption by inducing expression of dual specificity protein phosphatase (Dusp) 1 and 2, thus causing a cell-autonomous negative feedback loop in osteoclasts. In particular, these data were partially consistent with our data in that STAT5 overexpression did not affect the formation of multinucleated TRAP^+^ cells but suppressed pit formation by osteoclasts. In contrast to a previous report (46), IL-7 did not reduce the phosphorylation of Erk although it increased phosphorylated STAT5, even though cells were treated with RANKL at the same time (Figure 5B), suggesting that activation of STAT5 by IL-7 did not seem to affect phosphatases, such as Dusp1 and Dusp2, to regulate mitogen-activated protein kinase (MAPK) activity. That is, STAT5 activation *via* stimulation with IL-3 repressed RANKL-mediated osteoclast formation, whereas appropriate activation of STAT5 by IL-7 promotes osteoclastogenesis. This discrepancy is possibly due to the difference in the model system—human primary monocytes and monocytic and macrophage-like cell lines RAW264.7 versus bone marrow-derived macrophage-like cells—used to analyze osteoclast differentiation. However, we believe that our system showed osteoclast differentiation by mimicking conditions that are close to those in the body as well as the inflammatory environment, using human samples, even though we primarily used *in vitro* experimental systems. In this regard, it is important to investigate a monocyte-specific IL-7Rα knockout model to analyze whether IL-7 regulates osteoclast differentiation directly *in vivo*. We thus plan to analyze the regulation of osteoclast differentiation in animal models *in vivo* in the future. Another limitation was that factors that increase the expression of IL-7Rα in monocytes needed to be identified through transcriptome and proteome analyses. Although we investigated which factor(s) may control the expression of IL-7Rα on CD14^+^ monocytes, we did not determine such factors. It is believed that these factors could also be a target for therapeutic agents that block the differentiation of osteoclasts in inflammatory conditions, either alone or in combination with other inhibitors.

In conclusion, our findings highlight the notion that IL-7 is a genuine osteoclastogenic factor, inducing osteoclast formation *via* the IL-7/IL-7Rα signaling pathway in the absence or presence of trace amounts of RANKL, a finding that also suggests IL-7 may be an important mediator in bone loss under inflammatory conditions. Given that RA likely represents a heterogeneous group of conditions with a similar phenotype, we believe IL-7 may be a good alternative therapeutic target for a specific subgroup of RA patients, and for those where a RANKL inhibitor or TNF-α inhibitor is not effective. We also demonstrated that STAT5 is an important mediator of IL-7-induced osteoclast differentiation and suggest the possibility of using JAK/STAT5 as a therapeutic target for arthritis.

## Ethics Statement

This protocol was approved by the Institutional Review Board of Seoul National University Hospital (#1406-043-584). Human peripheral blood and synovial fluid were drawn from healthy volunteers and patients with RA after obtaining written informed consent in accordance with the Declaration of Helsinki.

## Author Contributions

SK and H-RK had full access to all data in the study and took responsibility for the integrity of the data, as well as for the manuscript. J-HK, JS, SK, and H-RK performed most of the experiments, data analysis, and manuscript preparation. SL, MS, S-KY, HS, J-HK, EL, YL, YC, W-HY, JK, W-UK, D-SL, and IK participated in data acquisition and analysis. All authors have read and approved the final manuscript.

## Conflict of Interest Statement

The authors declare that the research was conducted in the absence of any commercial or financial relationships that could be construed as a potential conflict of interest.

## Abbreviations

IL-7Rα, IL-7 receptor alpha (α) chain; RANKL, receptor activator of nuclear factor κB ligand; M-CSF, macrophage colony-stimulating factor; IL-1β, interleukin-1β; TNF-α, tumor necrosis factor-α; TRAP, tartrate resistant acid phosphatase; JAK, Janus kinase; STAT5, signal transducer and activator of transcription 5; PI3K, phosphoinositide -3 kinase; Erk, extracellular signal-regulated kinases; OPG, osteoprotegerin; PBMC, peripheral blood mononuclear cell; SFMC, synovial fluid mononuclear cell; NFATc1, nuclear factor of activated T-cells 1.
