# Corrigendum: Interleukin-7 Induces Osteoclast Formation *via* STAT5, Independent of Receptor Activator of NF-kappaB Ligand

**DOI:** 10.3389/fimmu.2017.01735

**Published:** 2017-12-07

**Authors:** Jin-Hee Kim, Ji Hyun Sim, Sunkyung Lee, Min A. Seol, Sang-Kyu Ye, Hyun Mu Shin, Eun Bong Lee, Yun Jong Lee, Yun Jung Choi, Wan-Hee Yoo, Jin Hyun Kim, Wan-Uk Kim, Dong-Sup Lee, Jin-Hong Kim, Insoo Kang, Seong Wook Kang, Hang-Rae Kim

**Affiliations:** ^1^Department of Anatomy and Cell Biology, Seoul National University College of Medicine, Seoul, South Korea; ^2^Department of Biomedical Laboratory Science, College of Health Science, Cheongju University, Cheongju, South Korea; ^3^Department of Biomedical Sciences, Seoul National University College of Medicine, Seoul, South Korea; ^4^BK21Plus Biomedical Science Project, Seoul National University College of Medicine, Seoul, South Korea; ^5^Department of Pharmacology, Seoul National University College of Medicine, Seoul, South Korea; ^6^Medical Research Institute, Seoul National University College of Medicine, Seoul, South Korea; ^7^Department of Internal Medicine, Seoul National University College of Medicine, Seoul, South Korea; ^8^Department of Internal Medicine, Chonbuk National University Medical School and Research Institute of Clinical Medicine of Chonbuk National University Hospital, Jeonju, South Korea; ^9^Department of Internal Medicine, Chungnam National University School of Medicine, Daejeon, South Korea; ^10^Department of Internal Medicine, The Catholic University of Korea, Seoul, South Korea; ^11^Department of Biological Sciences, College of Natural Sciences, Seoul National University, Seoul, South Korea; ^12^Department of Internal Medicine, Section of Rheumatology, Yale University School of Medicine, New Haven, CT, United States

**Keywords:** osteoclast, intereleukin-7, intereleukin-7 receptor alpha, STAT5, RANKL, monocyte

In the original article, there was a mistake in the legend for **Figure 2** as published. Here, the expression “SFMCs from healthy individuals” should be corrected to “SFMCs”. The correct legend appears below. The authors apologize for this error and state that this does not change the scientific conclusions of the article in any way.

**FIGURE 2** | Interleukin (IL)-7 induced osteoclast formation in synovial fluid mononuclear cells (SFMCs) from joint fluid of rheumatoid arthritis (RA) patients. SFMCs were cultured with M-CSF (20 ng/mL), RANKL (50 ng/mL), or IL-7 (2 ng/mL) for 10 days by replacing the medium at 3-day intervals with fresh cytokines as described in Figure 1 (left panel). To determine the effect of pretreatment with IL-7, SFMCs were cultured with IL-7 (2 ng/mL) for 3 days, then treated with M-CSF (20 ng/mL), RANKL (50 ng/mL), or IL-7 (2 ng/mL) for 7 days, replacing the medium as described above (right panel). TRAP staining and enumeration were performed as described in Figure 1. Representative images **(A)** and quantification **(B)** of TRAP^+^ cells at days 10 and 15 are shown. Results are representative of five independent experiments with five different donors. Bars represent the mean and *p* values were obtained using the unpaired two-tailed Student’s *t*-test. **(C)** Peripheral blood mononuclear cells were cultured on top of dentine disks in 96-well culture plates in the above condition for 30 days. Then, surface roughness was analyzed as described in Figure 1. Results illustrate three independent experiments (*n* = 3). Roughness parameter and the number of pits were analyzed as described in Figure 1. The graph represents the mean ± SEM and *p* values were obtained using the unpaired two-tailed Student’s *t*-test.

The original article was updated.

## Conflict of Interest Statement

The authors declare that the research was conducted in the absence of any commercial or financial relationships that could be construed as a potential conflict of interest.

