# Supplementary material for: Interleukin-7 Induces Osteoclast Formation via STAT5, Independent of Receptor Activator of NF-kappaB Ligand
Source: Front Immunol. 2017 Oct 20;8:1376. doi: 10.3389/fimmu.2017.01376 (PMC5655015; doi:10.3389/fimmu.2017.01376)
Supplement: Supplementary file 1 [file Table_1.DOCX]

**Supplementary table**

**Table S1**. PCR primers.

| Gene |  | Sequence |
| --- | --- | --- |
| Mouse Genes | | |
| *TRAP* | Forward | 5’- GAC-GAT-GGG-CGC-TGA-CTT-CA -3’ |
|  | Reverse | 5’- GCG-CTT-GGA-GAT-CTT-AGA-GT -3’ |
| *CathK* | Forward | 5’- ACG-GAG-GCA-TCG-ACT-CTG-AA -3’ |
|  | Reverse | 5’- GAT-GCC-AAG-CTT-GCG-TCG-AT -3’ |
| *CalcR* | Forward | 5’- GAC-AAC-TGC-TGG-CTG-AGT-G -3’ |
|  | Reverse | 5’- GAA-GCA-GTA-GAT-AGT-CGC-CA -3’ |
| *c-src* | Forward | 5’- CCA-GGC-TGA-GGA-GTG-GTA-CT -3’ |
|  | Reverse | 5’- CAG-CTT-GCG-GAT-CTT-GTA-GT-3’ |
| *NFATc1* | Forward | 5’- CTC-GAA-AGA-CAG-CAC-TGG-AGC-AT -3’ |
|  | Reverse | 5’- CGG-CTG-CCT-TCC-GTC-TCA-TAG -3’ |
| *GAPDH* | Forward | 5’- CTG-CAC-CAC-CAA-CTG-CTT-AG -3’ |
|  | Reverse | 5’- AGA-TCC-ACG-ACG-GAC-ACA-TT -3’ |
| Human Genes | | |
| *CathK* | Forward | 5’- ACC-GGG-GTA-TTG-ACT-CTG-AA -3’ |
|  | Reverse | 5’- GAG-GTC-AGG-CTT-GCA-TCA-AT -3’ |
| *RANK* | Forward | 5’- CGT-AGA-CCA-CGA-TGA-TGT-CG -3’ |
|  | Reverse | 5’- GTC-TGG-AAG-CTC-CCC-TGG-T -3’ |
| *RPS18* | Forward | 5’- GAT-GGG-CGG-CGG-AAA-ATA-G -3’ |
|  | Reverse | 5’- GCG-TGG-ATT-CTG-CAT-AAT-GGT -3’ |

*TRAP, tartrate-resistant acid phosphatase; CathK, cathepsin K; CalcR, calcitonin receptor; c-src, tyrosine-protein kinase SRC-1; GAPHD, glyceraldehyde 3-phosphate dehydrogenase; NFATc1, nuclear factor of activated T-cells 1. RANK, receptor activator of nuclear factor κB; RPS18, Ribosomal Protein S18*
