# Supplementary figures and images for: Interleukin-7 Induces Osteoclast Formation via STAT5, Independent of Receptor Activator of NF-kappaB Ligand
Source: Front Immunol. 2017 Oct 20;8:1376. doi: 10.3389/fimmu.2017.01376 (PMC5655015; doi:10.3389/fimmu.2017.01376)

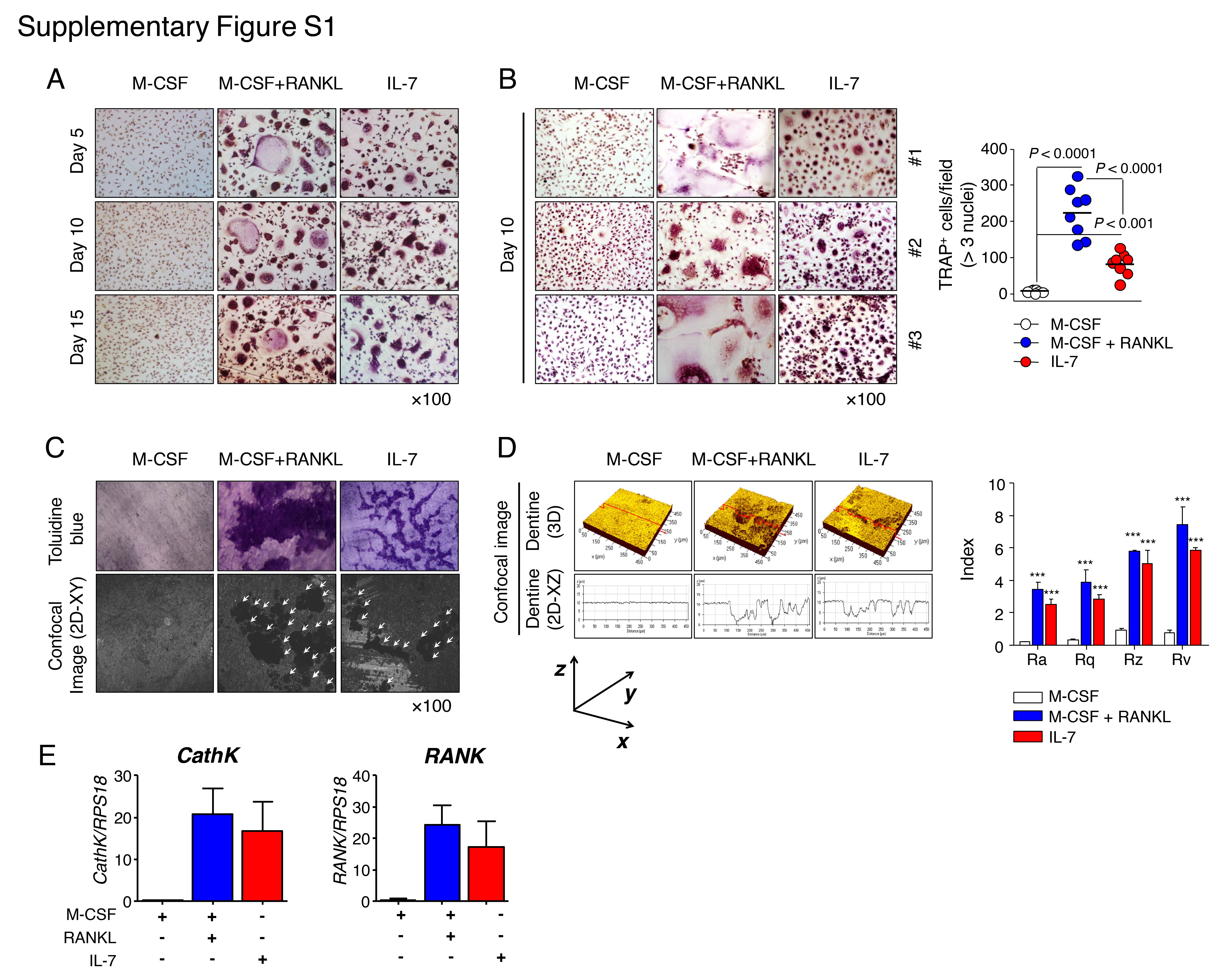

Supplement: Supplementary file 2 [file Image_1.JPEG]

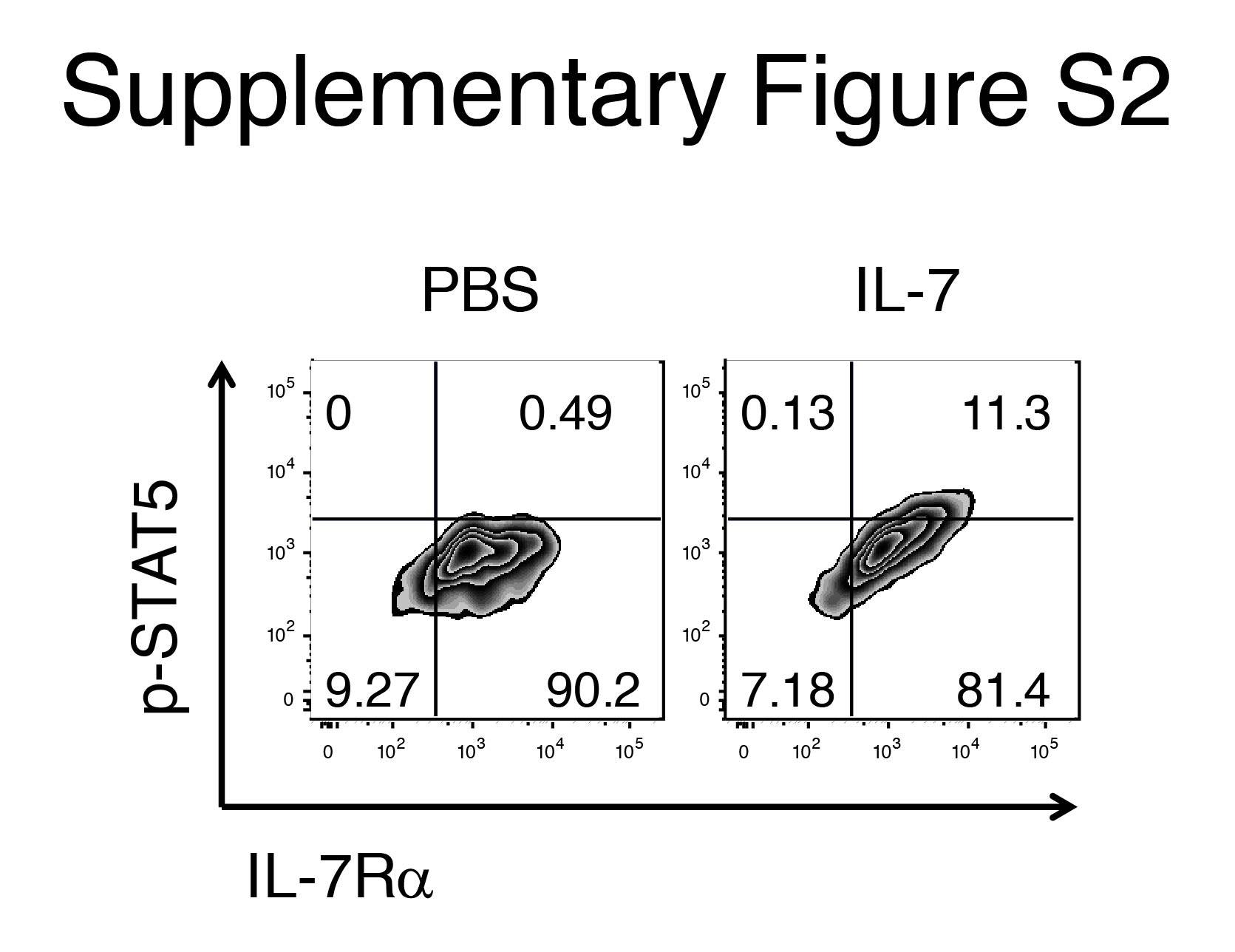

Supplement: Supplementary file 3 [file Image_2.JPEG]

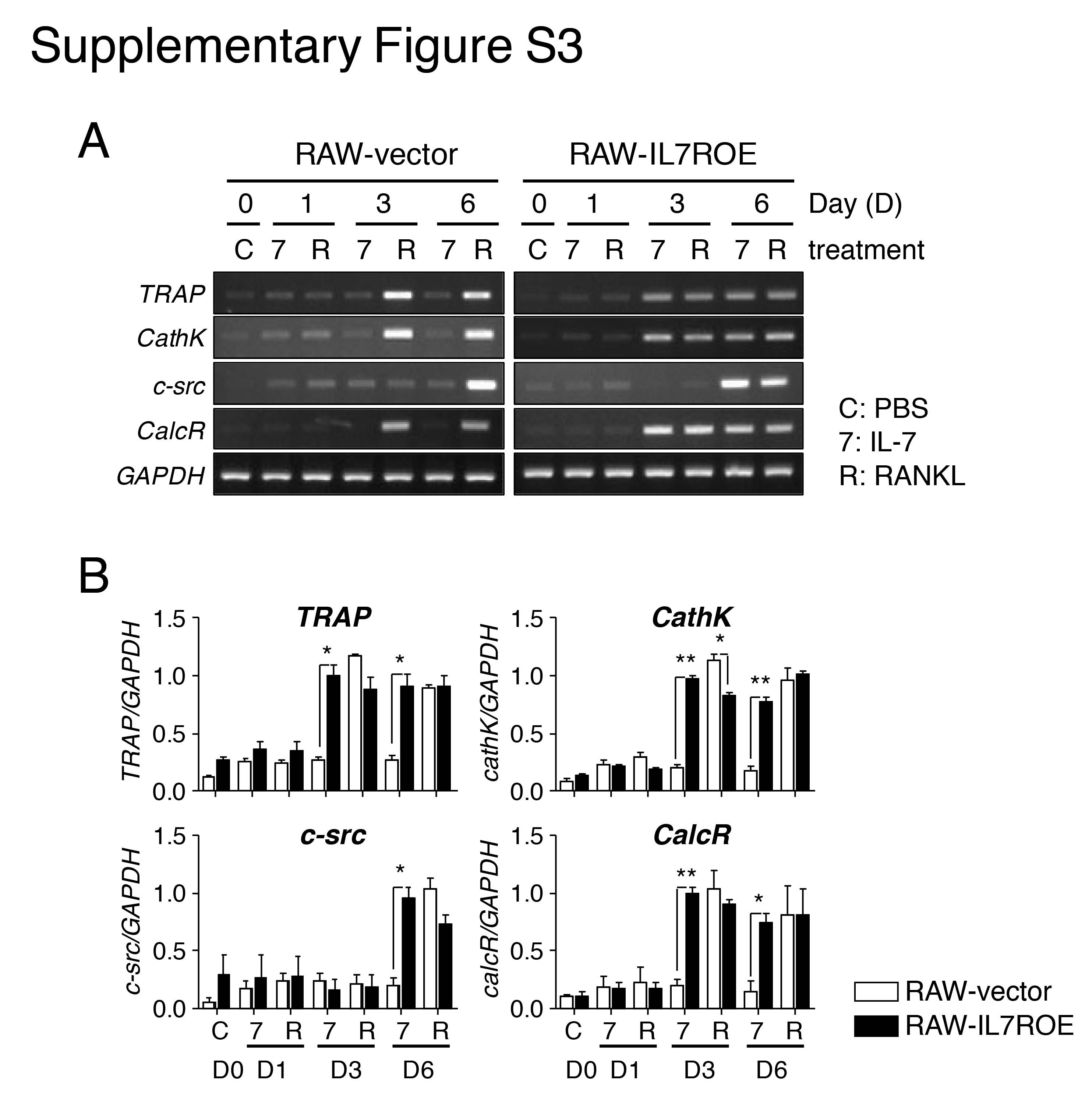

Supplement: Supplementary file 4 [file Image_3.JPEG]

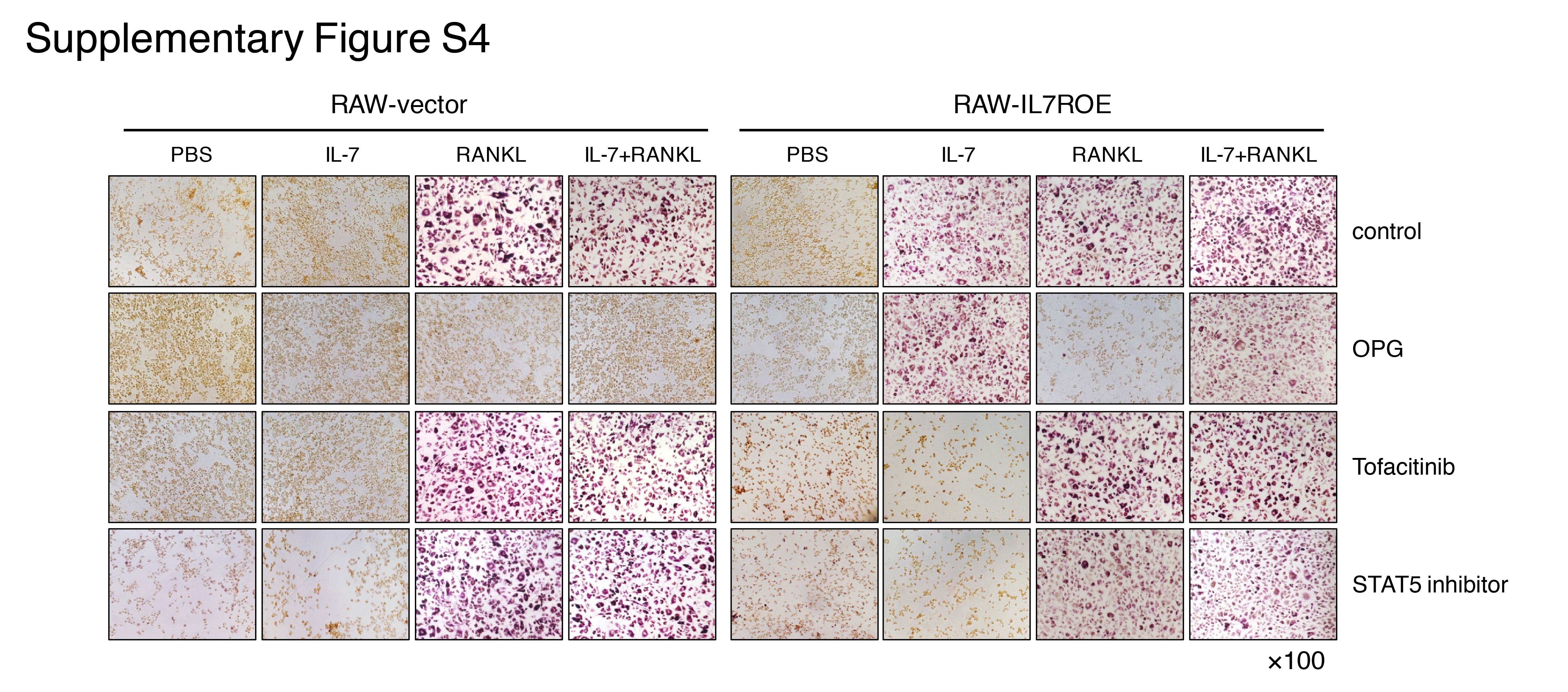

Supplement: Supplementary file 5 [file Image_4.JPEG]
